# Phytochemical analysis, antioxidant and in vitro β-galactosidase inhibition activities of *Juniperus phoenicea* and *Calicotome villosa* methanolic extracts

**DOI:** 10.1186/s13065-021-00781-y

**Published:** 2021-10-04

**Authors:** Ahmed Al-Mustafa, Mohammad Al-Tawarah, Mohammed Sharif Al-Sheraideh, Fatema Attia Al-Zahrany

**Affiliations:** 1 Department of Biological Sciences, Faculty of Science, Mutah University, Mutah, P.O. Box 7, Karak, 61710 Jordan; 2 Chemistry Department, College of Science, Imam Abdulrahman Bin Faisal University, Dammam, Saudi Arabia

## Abstract

**Background:**

*Juniperus Phoenicea* (JP) and *Calicotome Villosa* (CV) are used by Jordanian populations as herbal remedies in traditional medicine. Herein, the phytochemical contents of their methanolic extracts were analyzed and their antioxidant as well as in vitro anti- β-Galactosidase activities were evaluated; their effect on β-Galactosidase enzyme kinetics was evaluated and the thermodynamic of the enzyme was determined.

**Methods:**

The antioxidant activity of JP and CV crude methanolic extracts was evaluated using 1,1-diphenyl,2-picrylhydrazyl (DPPH) free radical scavenging and ferric reducing antioxidant power (FRAP) assays; however, the effect of the plants’ crude extracts on β-Galactosidase activity and kinetics was evaluated in vitro. Moreover, total phenolic, flavonoids, and flavonols content in plants’ extracts were determined and expressed in Gallic acid equivalent (mg GAE/g dry extract) or rutin equivalent (mg RE/g dry extract).

**Results:**

Phytochemical screening of the crude extracts of JP and CV leaves revealed the presence of phenols, alkaloids, flavonoids, terpenoids, anthraquinones, and glycosides. Flavonoids and flavonols contents were significantly higher in JP than in CV (p < 0.05)*.* Furthermore, an analogous phenolic content was detected in both JP and CV methanolic extracts (103.6 vs 99.1 mg GAE/g extract). The ability of JP extract to scavenge DPPH radicals was significantly higher than that of CV extract with IC_50_ = 11.1 μg/ml and 15.6 μg/ml, respectively. However, their extracts revealed relatively similar antioxidant capacities in FRAP assay; their activity was concentration dependent. The JP extract inhibited β—galactosidase enzyme activity with a significant IC_50_ value compared to CV extract; they exhibited their inhibitory activities at IC_50_ values 65 µg/ml and 700 µg/ml, respectively. Rutin revealed anti-β-galactosidase activity at IC_50_ = 75 µg/ml. The mode of inhibition of β-galactosidase by JP, CV, and rutin was non-competitive, mixed, and competitive inhibition, respectively. Thermodynamic and enzyme inactivation kinetics revealed that β-galactosidase has a half-life time of 108 min at 55 °C, activation energy of 208.88 kJ mol^−1^ and the inactivation kinetics follows a first-order reaction with k-values 0.0023–0.0862 min^−1^ and positive entropy of inactivation (∆*S*°) values at various temperatures, indicating non-significant processes of aggregation.

**Conclusions:**

The methanolic extracts of JP and CV possess anti-hyperglycemic and antioxidant activities with potential pharmaceutical applications.

## Introduction

Medicinal plants are used traditionally in developing countries as therapeutic agents in the treatment of chronic diseases [1]. They are one of the main sources of phytochemicals, like polyphenols, that have numerous pharmacological advantages as antioxidant, antiviral, anticancer, antimicrobial, antifungal, and antidiabetic remedies [2–6]. Jordan is a country with species richness and diversity of medicinal plants that are used in traditional medicine regardless of the economic or educational levels of the patients [7].

*Juniperus phoenicea* L. (JP), family *Cupressaceae,* is a shrub or a small tree growing in the coastal sites of the Mediterranean Region, in some Middle East countries, widely distributed in Europe, northern Africa, and the Canary Islands [8]. In Jordan, it is distributed throughout the South Mountain regions, in Tafileh and Shoubak woodlands at high altitudes of 1200–1700 m [9]. The leaves of JP are used in the form of decoction to treat diabetes, diarrhea, rheumatism, and as a diuretic against bronchopulmonary disease but a mixture of its leaves and berries is used as an oral hypoglycaemic agent [6, 7]. Abu-Darwish et al. [10] reported that the essential oils of JP, collected from the South of Jordan, are characterized by a high percentage of α-pinene content. Furthermore, the flavonoids myricitrin, quercetin, cosmosin, and quercitrin in addition to sterols, and hydrocarbons were isolated from the leaves of Egyptian JP [4, 11]. In fact, essential oils and extracts of JP were reported as antioxidants and antimicrobial agents [11–13]. Their hypoglycaemic and hypolipidemic effects as well as cytotoxic activity were also investigated [14–17].

However, *Calicotome* villosa (Poir.) Link subspecies intermedia, family *Fabaceae*, is a thorny shrub that can reach 2 m in height and has sharp terminations, trifoliate including oval leaves with yellow and grouped flowers [18]. It grows mostly in cool places and is very common in North Africa (Morocco and Algeria) and Spain [19]. The plant has been used in traditional medicine by Mediterranean populations for the treatment of furuncle, cutaneous abscess, chilblain, and as an antitumor agent [20–22]. The bioactivities of CV plant extracts were ascribed to their content of flavone glucosides, alkaloids, and anthraquinones in the aerial parts [18, 23, 24] as well as flavonols and alkaloids in the seeds [25].

Diabetes mellitus is one of the most common metabolic disorders due to lack of insulin production or the inability to control blood glucose by insulin leading to hyperglycemia [26]. Therefore, inhibition of β-galactosidase, an enzyme that hydrolyze glycosidic bonds in complex carbohydrates or glycoconjugates, influences glucose uptake by the cells and is a target for regulation of hyperglycemia [27]. Interestingly, the activity and thermal stability of β-galactosidase enzymes are influenced by diverse environmental factors (temperature, pH, and reaction medium) which can strongly affect the specific three-dimensional structure or spatial conformation of the protein [28], thereby will affect the enzyme’s physiological function. Several biotechnological approaches were used to study enzyme deactivation, which forms a major and important constraint in estimation of the enzymes’ thermodynamic parameters; this will lead to understanding the probable denaturation mechanism in enzymatic processes [29].

The current study aimed to analyze the phytochemical content of JP and CV methanolic extracts with evaluating their antioxidant and anti- β-Galactosidase activates as well as their role in kinetic and thermodynamic parameters of the enzyme*.*

## Materials and methods

### Chemicals

β-Galactosidase from *Aspergillus oryzae* (CAS Number: 9031-11-2), Folin-Ciocalteu phenol, 2,4,6-tripyridyl-s-triazine (TPTZ), 2, 2-diphenyl-2-picrylhydrazyl (DPPH), Rutin and Gallic acid were obtained from Sigma-Aldrich. *ο*-Nitrophenyl-β-D-Galactopyranoside (CAS Number: 369–07-3) was obtained from ACROS ORGANICS. Other chemicals and solvents were used of analytical grade.

### Plant material collection and extraction

JP and CV fresh leaves were collected in April from Al-Shoubak region in the southern part of Jordan (30°31′4.52"N, 35°33′25.59"E). The plants’ samples were identified morphologically by a plant specialist in the Department of Biology, Faculty of Sciences/University of Mutah. Voucher specimens were kept at the Laboratory of plant science, Department of Biology/Mutah University.

The extracts were prepared according to [30] with some modifications. The JP and CV leaves were air-dried in shade and (30 g) of the pulverized plant samples were extracted in 300 ml of methanol (99%) for 48 h under continuous shaking. The resulting extracts were filtered through a Whatman paper (No. 4) and concentrated *in vacuum* at 40 °C using a rotary evaporator (Buchi R-215, Switzerland). The methanol extracts were kept at 4 °C in the dark until further use.

### Phytochemical screening

#### Qualitative analysis

The qualitative tests of extracts’ phytochemical constituent were carried out according to [31, 32]; it based on the observed visual color change of methanol extracts of JP and CV with added reagents. The analysis was performed to detect presence of alkaloids, flavonoids, saponins, terpenoids, phenols, anthraquinones, tannins, glycosides, and anthocyanins in plants’ extracts. The results are expressed as ( +) for the presence and (-) for the absence of phytochemicals.

#### Quantitative analysis

##### Determination of total phenols

The total phenolic content of JP and CV was assayed using the Folin-Ciocalteu reagent with gallic acid as a standard [33]. 0.2 mL of (10 mg/ml) extract was mixed with Folin-Ciocalteu phenol reagent (1.5 mL). After 5 min, 6% sodium carbonate (1.5 mL) was added, and the mixture was allowed to stand at room temperature for 90 min. The absorbance was measured at 725 nm and the results were expressed as mg of gallic acid equivalent per gram of extract (mg GAE/g). The assays were performed in triplicates.

##### Determination of total flavonoids

The total flavonoid content in plant methanolic extract was estimated according to [34] with some modifications. 1 ml of tested extract (10 mg/ml) was added to a volumetric flask containing 4 ml of distilled water. 0.3 ml of 5% NaNO_2_ solution was added to the preparation which left for 6 min, then 0.3 ml of 10% AlCl_3_ solution was added and kept for another 6 min. To this reaction mixture, 4 ml of 1 M NaOH solution and 0.4 ml water were added to make up a final volume of 10 ml. The reaction mixture was mixed well and allowed to stand for 15 min, after which absorbance was recorded at 510 nm. Total flavonoid content was expressed as mg rutin equivalent (RE)/g plant sample. Tests were performed in triplicates.

##### Determination of total flavonols

The total flavonols contents of plants extracts were determined following the procedure reported by Abdel-Hameed [35] using rutin as a standard. Briefly, 0.5 ml of methanolic plant extract (10 mg/ml) was mixed with 0.5 ml (20 mg/ml) AlCl_3_ and 1.5 ml (50 mg/ml) sodium acetate. After 2.5 h on incubation at 25 °C, the absorbance of the reaction was measured at 440 nm (UV/VIS Spectrophotometer, Novaspec Model 80–2088-64, Pharmacia Bio-tech, UK). Triplicate determinations were carried out.

### Antioxidant activity

#### Determination of DPPH radical scavenging activity

The free radical scavenging capacity of extracts was determined using the method described by Deng et al. [36] with gallic acid as a reference. Briefly, 50 µl of each extract was added to 950 µL of methanolic DPPH solution (A_517nm_ 0.74). The preparations were incubated for 30 min at room temperature in dark and their absorbance was measured at 517 nm. Antioxidant activity of the plant extracts was calculated as follows:

$${\rm{I }}\left( \% \right){\mkern 1mu} = {\mkern 1mu} \left( {\left( {{{\rm{A}}_{{\rm{blank}}}} - {\rm{ }}{{\rm{A}}_{{\rm{sample}}}}} \right)/{\rm{ }}{{\rm{A}}_{{\rm{blank}}}}{\rm{}}} \right) \times 100$$

where I inhibition in DPPH absorbance, A_blank_ is the absorbance of the control reaction (containing all reagents except the plant extract), and A_sample_ is the absorbance of the tested plant extract. Extract concentrations providing 50% inhibition (IC_50_) are calculated from the plot of inhibition (%) against extract concentration and compared with the IC_50_ of Gallic acid tested following the same procedure. Samples were carried out in triplicates.

#### Ferric Reducing Antioxidant Power (FRAP) Assay

Ferric reducing antioxidant power of methanolic extracts of plants was performed as described previously [37]. In the FRAP assay, 300 mM acetate buffer, pH 3.6, 20 mM ferric chloride and 10 mM 2,4,6-tripyridyl-s-triazine (TPTZ) were dissolved in 40 mM HCl (10:1:1 ratio). 0.1 ml of different plant extract concentrations was added to 0.9 ml of FRAP working reagent to have a final concentration of 5, 10, 15, 20, and 25 µg/ml. The absorbance of the reaction mixture was measured at 593 nm after 10 min of incubation at 37 ^o^ C. FeSO_4_.7H_2_O (20 mM) was used for establishing the calibration curve. The change in absorbance was calculated as FRAP value (µM) by relating the ratio of A_593 nm_ of the test sample to that of the standard solution of known FRAP value (100 µM) using the following Equation.

FRAP = (A_593nm_test sample/A_593nm_test standard) FRAP value of standard (µM).

#### β -Galactosidase activity assay

The β-galactosidase activity was measured as described by [38] with slight modification. 2-Nitrophenyl-β-D-Galactopyranoside (ONPG) was used as a substrate, which is hydrolyzed by β—Galactosidase to release 2-nitrophenyl (a colored agent, λmax 410 nm). The reaction mixtures contained 20 µmole of ONPG dissolved in 4 ml of 0.1 M citrate–phosphate buffer (pH 4.5) and 40 μl of β -Galactosidase solution (0.2 U/mL). After incubation for 20 min at 30˚C, the reaction was stopped by adding 1 ml of 1 M Na_2_CO_3_. The absorbance of the resulting product was measured at 420 nm. One unit of enzyme activity was defined as the amount of enzyme which liberated 1µmole of O-Nitrophenol per minute under assay conditions.

#### Determination of enzyme kinetics

To investigate the kinetics of the reaction catalyzed by β-galactosidase, the reaction velocity versus different substrate concentrations was studied according to [39]. The double reciprocal linear plot was applied to determine V_max_, K_M_ and to estimate reaction constants. Ki calculation depends on the mode of inhibition by comparing the "apparent" values of V_max_ and K_m_ for an enzyme in the presence and absence of an inhibitor.

#### Determination of β-Galactosidase relative activity (%)

The effect of JP and CV methanolic extracts as well as rutin, at different concentrations (16–800 µg/ml), on β-galactosidase activity was determined according to a standard assay test [38]. The relative activity (%) of the β-galactosidase was related to enzyme activity in absence of the effectors according to the following formula:

$${\text{Relative Activity }}\left( \% \right) = \, \left[ {\left( {{\text{Enzyme activity without}} - {\text{ Enzyme activity with extracts}}} \right)/{\text{ Enzyme activity without}}} \right] \, \times { 1}00$$

### Effect of reference sugars on β-galactosidase activity

The effect of glucose, galactose, fructose, and acarbose on β-galactosidase activity was evaluated at different concentrations that range from 80 µg/ml to 4000 µg/ml using ONPG as a substrate and under the same conditions used to study the enzyme activity.

### Thermal β-galactosidase inactivation studies

The thermal inactivation of β-galactosidase was determined at different time intervals and incubation temperatures. The enzyme solution was incubated in sealed tubes at 50, 55, 60, and 65 °C for 3 h in a thermostatically controlled water bath. One tube of β-galactosidase was withdrawn at each time interval, immediately immersed in an ice bath and enzyme activity was determined as described earlier. The activity after 1 min of heating-up time (t = 0) was the initial activity, thereby eliminating the effects of heating-up.

### Estimation of β-galactosidase kinetic and thermodynamic parameters

It is generally accepted that the thermal inactivation of several enzymes including β-galactosidase is a first-order reaction, and the reaction occurs at one inactivation rate (*k*-value) in a single step, therefore kinetic and thermodynamic parameters were determined as described in previous studies [29, 39].

### Statistical analysis

Results are expressed as a mean ± standard deviation. Differences between the means were determined by one-way analysis of variance (one-way ANOVA). A difference in the mean values of *p* < 0.05 was considered statistically significant. Also, IC_50_ values were determined using a nonlinear regression curve with the Microsoft Excel 2016.

## Results

### Extract yield and phytochemical screening

The total yields of JP and CV leaves methanol extracts were 22.83 and 14.37% (w/w), respectively. Their leaves’ extracts contained various secondary metabolites such as phenols, alkaloids, flavonoids, terpenoids, anthraquinones, and glycosides (Table 1). Furthermore, the total phenolic, flavonoids, and flavonols contents in JP extract were 103.6 mg GAE/g, 101.1 mg RE/g, and 30.7 mg RE/g), respectively; meanwhile, they were 99.1 mg GAE/g, 61.6 mg RE/g, and 19.2 mg RE/g in case of CV extract, respectively (Table 2). The flavonoids and flavonols contents were significantly higher in JP crude extract compared to CV extract (p˂ 0.05).

**Table 1:** Screening of phytochemicals in *J. phoenicea* and* C. villosa* leaves methanolic extract

No	Phytochemicals	*J. phoenicea*	*C. villosa*
1	Alkaloids	+	+
2	Anthocyanins	+	+
3	Flavonoids	+	+
4	Phenols	+	+
5	Saponins	+	+
6	Tannins	+	+
7	Terpenoids	+	+
8	Glycosides	+	+
9	Anthraquinons	+	+

**Table 2:** Total phenolic, flavonoids, and flavonols contents in *J. phoenicea* and* C. villosa* leaves methanol extracts

Extract	Total phenols (mg GAE/g extract)	Total flavonoids (mg RE/g extract)	Total flavonols (mg RE/g extract)
*J.phoenicea*	103.6 ± 0.006^a^	101.1 ± 0.016^b^	30.7 ± 0.082^d^
*C.villosa*	99.1 ± 0.013^a^	61.6 ± 0.0075^c^	19.2 ± 0.088^d^

Result expressed as mean ± SD of n = 3. Values in the same column with different letters are statistically significant at p < 0.05

### Antioxidant activity

Evaluating the ability of tested crude extracts and the gallic acid standard at (2 µg/ml) to scavenge generated DPPH radical revealed percentage of inhibitions about 62.41%, 10%, and 8.28%, respectively. The order of antioxidant potency was Gallic acid < JP < CV and in a concentration dependent manner. The IC_50_ values for JP, CV and gallic acid in DPPH assay were 11.1 µg/ml, 15.6 µg/ml, and 1.3 µg/ml, respectively; they were interpreted from extrapolated regression curve of % inhibition in DPPH radical against used sample concentrations (Table 3).

**Table 3:** DPPH antioxidant activity of *J. phoenicea* and *C. villosa* leaves methanolic extracts

Extract	DPPH radical scavenging activity (IC_50_ = µg/ml)
*J.phoenicea*	11.10 ± 0.015^a^
*C.villosa*	15.6 ± 0.019^b^
Gallic acid	1.32 ± 0.011 ^c^

Result expressed as mean IC_50_ values ± SD from three independent experiments. Values with different letter are significantly different compared to control (P < 0.05)

**Fig. 1:**
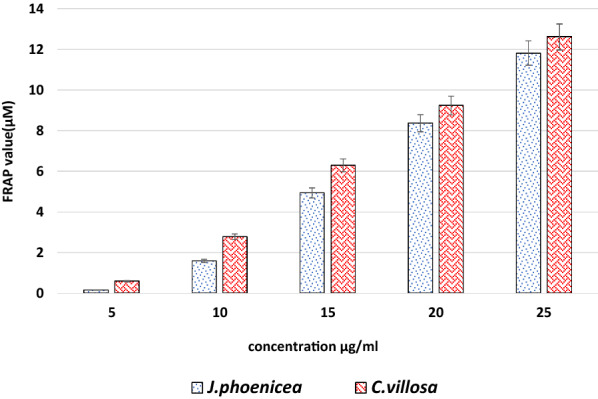
FRAP value of *J. phoenicea* and *C. villosa* extracts. Each value represent means ± SD (n = 3)

Furthermore, the effect of plants’ extracts on ferric ion reduction in FRAP test as indication of their antioxidant potential revealed that both extracts are considered as primary antioxidant agents with non-significant differences in their effect at tested concentrations, represented by their FRAP values (µM) (Fig. 1). Their effect in ferric ion reduction was concentration dependent with a positive correlation between ferric reducing capacity and the concentration of plant extract, JP (R^2^ = 0.9954) and CV (R^2^ = 0.9825).

### Determination of enzyme kinetics

**Fig. 2:**
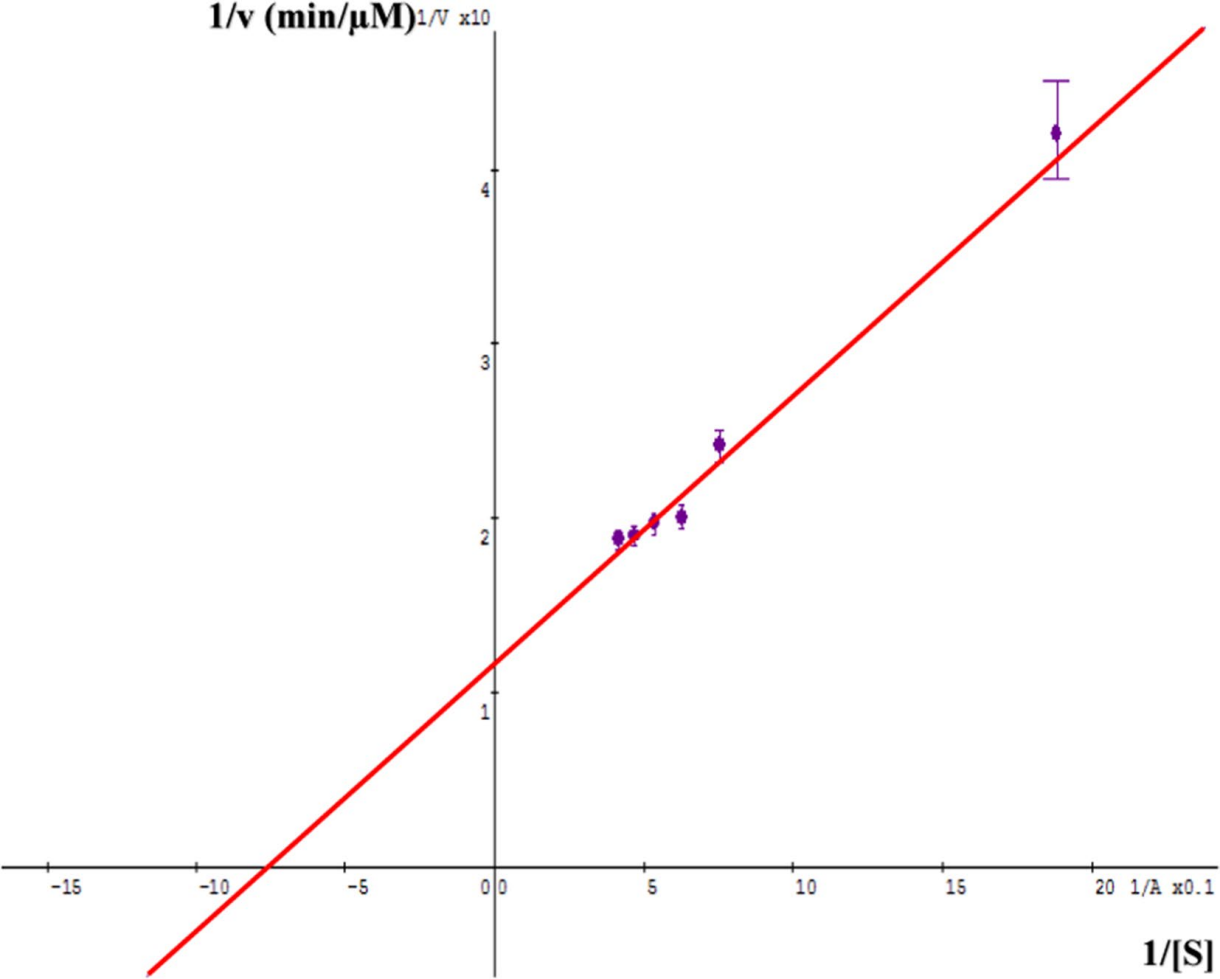
Lineweaver–Burk plot of (1/ v) of β-galactosidase vs (1/ ONPG). Mean ± SD (n = 3)

Determination of the β-galactosidase, from *Aspergillus* oryzae, enzyme kinetics was interpreted based on its ability to hydrolyze ONPG substrate at pH 4.5 and 30˚C. The enzyme’s kinetic parameters Km and Vmax were obtained by a typical double reciprocal Lineweaver Burk plot (Fig. 2) which were 1.310 ± 0.091 mM and 85.344 ± 0.028 mU, respectively.

### Effect of *J. Phoenicea,* and *C. villosa* on β-Galactosidase Activity

**Fig. 3:**
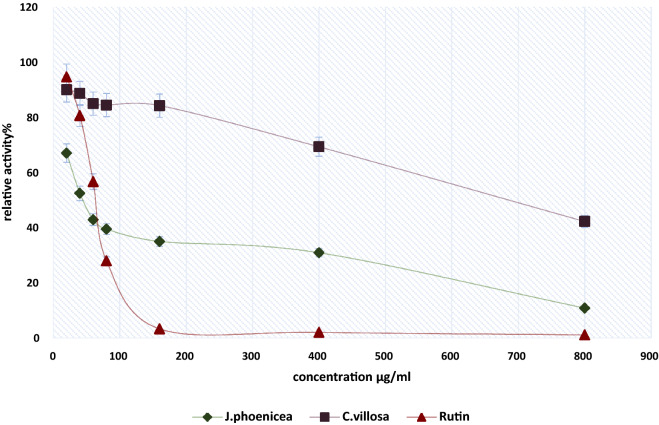
Relative activity (%) for β-galactosidase from A. *oryzae* in presence of *J. phoenicea*, *C. villosa*, and rutin, using ONPG as a substrate. Mean ± SD (n = 3)

**Fig. 4:**
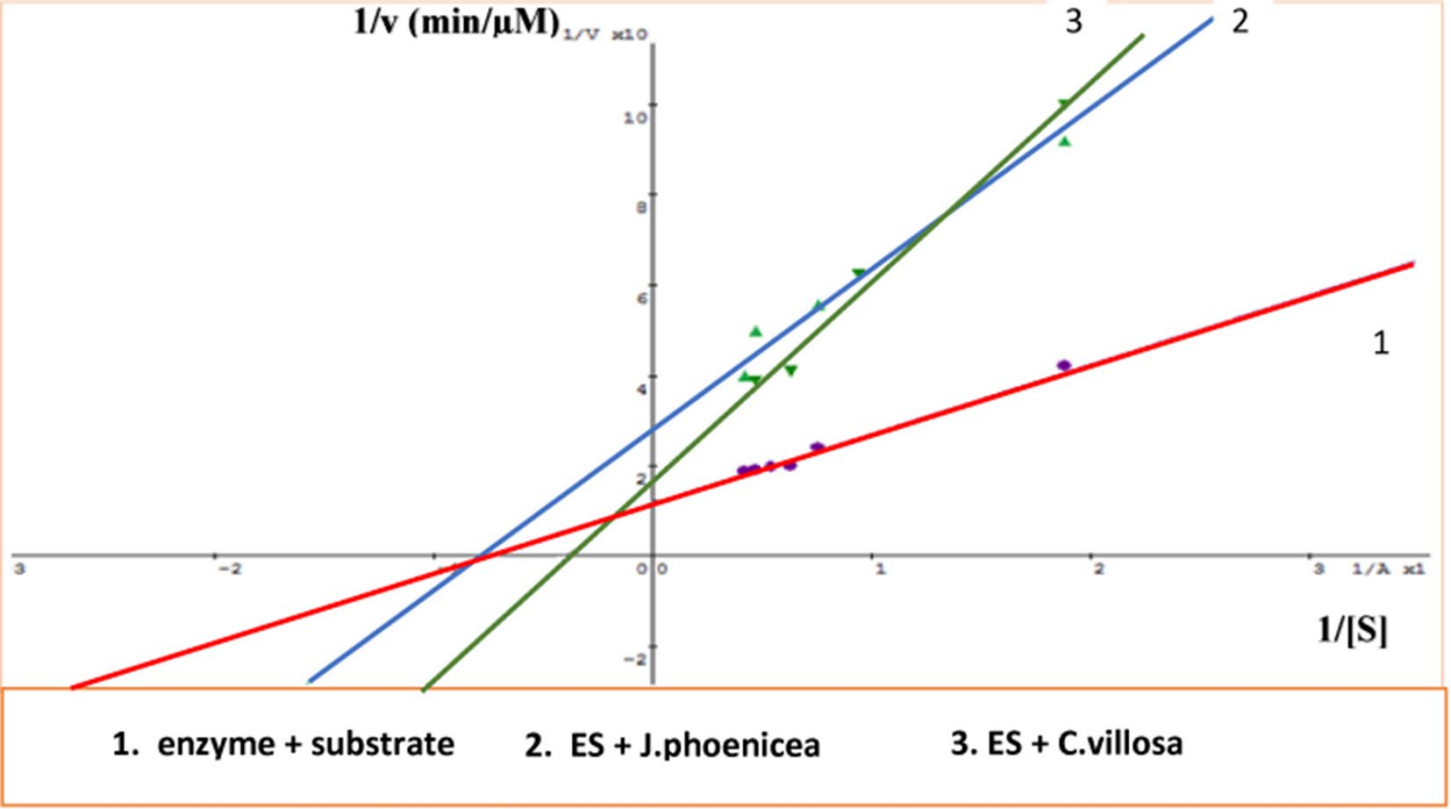
Inhibition of β-galactosidase by *J. phoenicea*, *AND C. villosa*, in the presence of various concentrations of ONPG as a substrate. Mean ± SD (n = 3)

The effect of JP and CV on the activity of β-galactosidase was determined by performing the standard assay procedure at different concentrations from 16 µg/ml to 800 µg/ml. The enzymatic activity was gradually decreased with increasing concentrations. Results of the percentage of relative activity (%) for the β-galactosidase in the presence of JP, CV, and rutin are shown in Fig. 3. JP extract inhibited β-galactosidase in a non-competitive way while that of CV extract was in mixed-inhibitory manner with IC_50_ values of 65 and 700 µg/ml, respectively (Fig. 4). Their effect on the kinetic parameters (Vmax, Km, and Ki) of β-galactosidase are shown in (Table 4).

**Table 4:** Kinetic values of β-galactosidase from *A. oryzae* in the presence of *J. phoenicea* and *C. villosa* extracts and Galactose

Inhibitor	Km (mM)	Vmax (mU)	Ki (mg/ml)	Mode of inhibition
Control	1.311 ± 0.091 ^a^	85.35 ± 0.028^a^	0	Normal
*J. phoenicea*	1.256 ± 0.089 ^a^	35.29 ± 0.033 ^b^	1.416 ± 0.058	Non-competitive
*C. villosa*	2.581 ± 0.034 ^b^	58.64 ± 0.015 ^c^	1.537 ± 0.039	Mixed
Galactose	2.770 ± 0.072 ^c^	55.55 ± 0.024 ^d^	1.866 ± 0.052	Mixed

Results are means ± SD (n = 3). Values in the same column with different letters differ significantly at p < 0.05

### Effect of reference sugars on β-galactosidase activity

**Fig. 5:**
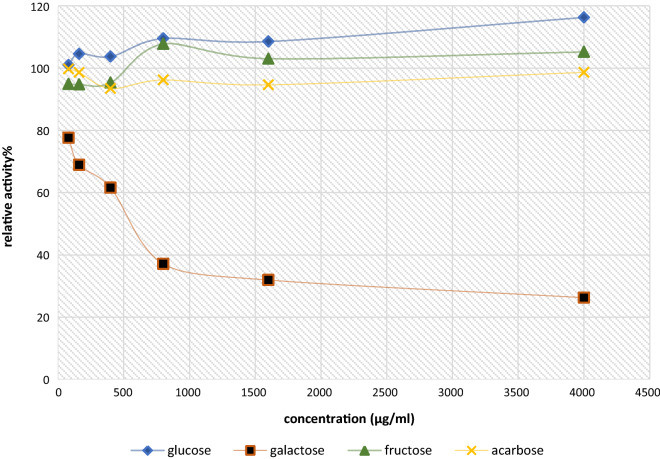
β-galactosidase relative activity in the presence of glucose, galactose, fructose and acarbose. Result expressed are mean of three independent experiments

The β-galactosidase activity in term of hydrolysis rate to the used ONPG substrate indicated a significant enhancement in enzyme activity in presence of glucose at concentrations over 1500 µg/ml, while the enzyme activity was inhibited significantly in the presence of galactose; a gradual decreased in the enzyme activity (23%, 39%, and 63%) was noticed at 80 µg/ml, 400 µg/ml, and 800 µg/ml of galactose, respectively. However, fructose and acarbose did not affect β-galactosidase at concentrations up to 4000 µg/ml (Fig. 5).

### Thermodynamic analysis for β-Galactosidase thermal inactivation

**Fig. 6:**
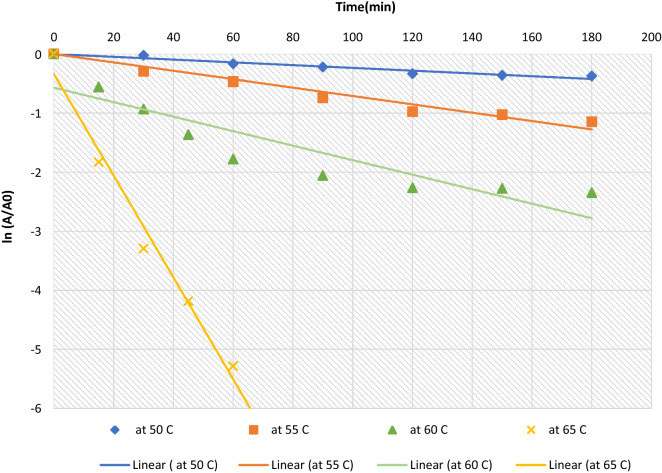
Thermal deactivation of β-galactosidase from *A*.* oryzae* at various temperatures using ONPG as a substrate. Result expressed are mean of three independent experiments

The experimental enzyme inactivation was performed at 5 °C temperature intervals (50, 55, 60, and 65 °C). The best fit of data is obtained when Ed = 208.88 kJ mol^−1^. The rate constant (kd) of β-galactosidase inactivation obtained are presented in Table 5; they were calculated from the plot of semi-natural logarithmic curve of residual activity versus time (Fig. 6). Similarly, the half-life (t_1/2_) for the enzyme to lose its 50% activity was estimated using the equation:

$${\text{t}}_{{{1}/{2}}} = \, 0.{693}/{\text{Kd}}$$

**Table 5:** Kinetic and thermodynamic parameters of β-galactosidase inactivation

Temp (°C)	Kd (min-1)	R2 (%)	t1/2 (min)	D (min)	∆H (kJ/mol)	∆G (kJ/mol)	∆S (J/(mol. K))
50	0.0023	94.39	301.30	1001.12	121.84	91.026	95.37
55	0.0064	96.49	108.28	359.78	121.80	89.644	98.00
60	0.0123	81.88	56.34	187.20	121.76	89.201	97.74
65	0.0862	98	8.04	26.71	121.72	85.068	108.39

**Fig. 7:**
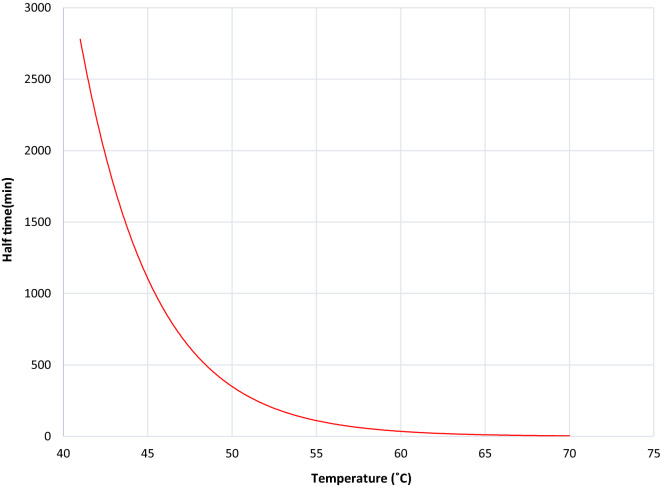
Half-life of β-galactosidase from A. *oryzae* as influenced by temperatures

Moreover, the plot of half-life in minutes is expressed as a function of temperature as represent in Fig. 7. Also, the D value was calculated using the relationship = ln_10_/Kd, and the z value was calculated and reported to be equal to 10 °C.

Table (5) shows the thermodynamic parameters of the enthalpy of denaturation (∆H°) for the enzyme at 50 to 65 °C, which were in a range of 121.84 to 121.72 kJ/mol. There was a decreasing trend with the increase in temperature. The free energy of thermal denaturation (∆G°) for β-galactosidase was 91.025 kJ/mol at 50 °C, which decreased with the increase in temperature to 85.068 kJ/mol at 65 °C. While the calculated entropy of inactivation (∆S°) showed positive values at each experimental temperature, which indicates that there are no significant processes of aggregation; otherwise if this had been happened, the values of (∆S°) would have been negative.

## Discussion

Natural products obtained from the medicinal plants encompass complex phytochemicals that are applied in several medical applications as anticancer, antidiabetic, and antioxidant agents. Furthermore, these chemicals are used in food industry and biotechnology due to their polyphenolic content [40–44]. Previous research, describe the potential use of *Juniper* species as sources of secondary metabolites, as antioxidant [45], antimicrobial [15, 16] or hypoglycaemic and hypolipidemic agents [46].

In this study, the qualitative and quantitative evaluation of the phytochemical constituents of JP and CV leaves extracts approved the presence of various secondary metabolites such as alkaloids, glycosides, polyphenols, saponins, terpenes, and anthraquinones, which confirms the medicinal importance of these plants. This finding is in accord with previous works [6, 18]. The methanol was used to extract secondary metabolites in JP and CV because of its capability to extract polar as well as nonpolar molecules and as recommended by [6]*.* This result agreed with previous work [6, 33, 47], which showed that methanol was a good solvent for the extraction of phenolic compounds from JP. It was documented that the highest phenolic content, for leaves and berries, was achieved by ethanol (35.4 and 35.8%, respectively), followed by methanol (26.2 and 16.4%, respectively) [6]. In this study, the total yield of methanol extracts of JP leaves was 22.83%, which was analogous to that reported in [6]. The total phenolic, flavonoids, and flavonols contents were higher in extract from leaves of JP than from CV. The variation in the phenolic yields between plant species as well as within the plant parts may be attributed to polarities of different compounds in the parts as well as to geographical location [48].

The amounts of total phenolics of both leaves of JP and CV were within the values ranged from 52 ± 1 to 217 ± 2 g GAE/kg of dry material, previously reported [6]. Consequently, the reporting of the high amount of phenols, flavonoids, and flavonols contents of JP leaves extracts could be used as an important species used in the food, and pharmaceutical industry.

The evaluation of different bioactivities of *JP and* CV crude extracts revealed that the JP methanolic extract possessed a high scavenging capacity for the generated DPPH^·^ radical at (IC_50_ = 11.10 ± 0.015 µg/ mL) than CV extract (IC_50_ = 15.6 ± 0.019 µg/ mL). This could be attributed to significant amounts of flavonoids present in JP. DPPH radical scavenging of JP extract showed 10 times less efficacy to the gallic acid standard reference (IC_50_ = 1.32 ± 0.011). The antioxidant activity of JP and CV leaves was within the results shown in earlier literate [6, 49]. The scavenging effect of the extracts against DPPH radicals and FRAP is related to the electron transfer/donating ability. Presence of flavonoids in JP and CV extracts as important phenolic compounds exert several antioxidant activities and ensuring free‐radical scavenging activity and protection against oxidative stress [50]; it confirms the ability of both extracts from JP and CV to scavenge the DPPH radicals.

Furthermore, JP extract exerts a higher activity as antioxidant than CV extract in FRAP assay; it might be attributed to the high phenolic content of JP extract. The availability of the phenolic hydrogens which behave as hydrogen donating radical scavengers can be easily predicted for antioxidant activity [51]. Therefore, polyphenols, depending on their precise structure and the proximity or adjacency of hydroxyl groups, have a metal-chelating potential. Thus, polyphenols may have the possibility of chelating metal ions and preventing iron- and copper- to initiate radical species [52, 53]. The reducing power of the extracts is consistent with the studies carried out on the aerial part of *A. longa* and *A. indica* [54–56], which indicated that they have a reducing power.

Diabetes is characterized by high blood sugar levels, which can lead to serious complications, so maintaining near-normal levels of glycemia is a goal of treating patients with diabetes [57]. The ability of JP and CV extracts to inhibit β–galactosidase in a concentration dependent manner might have a role in controlling blood glucose level. It was observed that the effect of JP extract was significantly higher than CV extract on the enzyme activity with IC_50_ = 65 µg/ml compared to 700 µg/ml, respectively. Previously it was reported that the more maceration of sample in polar solvents such as methanol, it will be a promising source for the reduction of postprandial glucose [57]. This finding is consistent with previous studies which demonstrated that some medicinal plant extracts, such as 75% aqueous -methanolic extract of propolis, inhibit α-glucosidase with an IC_50_ of 7.24 ± 1.16 µg/mL. The inhibitory effect of medicinal plants against β-Galactosidase activity showed various IC_50_ values range from 50 µg/ml to 5 mg/ml in different studies [58, 59]. Moreover, it was documented that galactose has an inhibitory effect on the activity of β-Galactosidase [60, 61]; a result which is on line with our finding on influence of galactose supplementation on the hydrolysis rate of the enzyme with IC_50_ of 600 µg/ml.

The effect of glucose on β-Galactosidase, demonstrated significant activation of hydrolysis rate at concentrations exceeded 1.5 mg/ml. This outcome confirms the hypothesis that glucose binds to the enzyme at a site other than the active one, in addition to affecting the affinity of the enzyme for the substrate [62]. Furthermore, the inability of fructose and acarbose to influence the enzyme catalytic activity was documented previously by different literatures [63].

As the kinetic parameters are important for studying β-Galactosidase enzyme behavior in presence of different factors, the mode of inhibition was obtained by a typical double reciprocal Lineweaver–Burk plot. Therefore, the results showed that the maximum velocity (V_max_) decreased in the presence of the extracts of JP as well as CV and galactose, but the Km increased by CV and galactose or slightly decreased by JP extract which indicating mixed inhibition and non-competitive inhibition, respectively. The mixed competitive inhibition indicates that the inhibitor can bind to the free enzyme or to the Enzyme–Substrate complex other than the catalytic site, which results in the decrease of Vmax [64]. Otherwise, a non-competitive inhibitor binds to a different site that may be a site other than the active site of the enzyme, due to a change in the chemical structure of the enzyme; therefore, it can block the binding of substrate or stopping of enzymatic activity. These results are consistent with previous studies that revealed inhibitory activity of galactose on β-Galactosidase enzyme [61].

To study the thermal stability of β-Galactosidase, the enzyme was tested at different temperatures (50 to 65 °C). Although β-Galactosidase was stable at 45 °C during the test period (3 h), its activity decreased at temperatures ≥ 50 °C. Inactivation of β-Galactosidase involves changing in the secondary, tertiary, or quaternary structure of a protein, without breaking covalent bonds [65]. The plots of residual activity vs. incubation time for the enzyme were linear, with R^2^ > 81%, which indicated that the inactivation could be expressed in terms of first-order kinetics in the temperature range of 50–65 °C. The kinetic enzymatic modeling of thermal inactivation and the determination of its thermodynamic parameters were analyzed at changeable temperatures and various conditions by [66]. The determinations of half-life (t_1/2_) are more accurate and reliable especially when computing the stability properties of an enzyme at different temperatures [67]. Thus, with increasing temperature, the t_1/2_ and D values were decreased, while the first-order thermal deactivation rate constant (kd) was increased. The results explored that the enzyme is less thermostable at higher temperatures, which led to a higher rate constant [68]. The z value of β -galactosidase, calculated from the slope of the graph between log D vs. temperature, was 10 °C. In general, the high magnitudes of z-values mean more sensitivity to the duration of heat treatment and lower z-values mean more sensitivity to increase in temperature [69].

The activation energy (Ed) of the thermal inactivation mechanism, which is the essential quantity to determine the thermodynamic parameters for thermal stability, is equal to 208.88 kJ mol^−1^ which is comparable to previous results on the inactivation of β-galactosidases from different strains of *Streptococcus thermophilus* and *Lactobacillus bulgaricus;* it was ranged from 200 to 215 kJ mol^−1^, indicating a high amount of activation energy needed to initiate denaturation of the enzyme [29]. Variations in activation enthalpy (∆H), activation entropy (∆S), and Gibbs free energy (∆G) were calculated for the different temperatures as listed in Table 7. Positive ΔH values indicate that enzyme inactivation is an endothermic process [70]. The high values of ∆H° resulted from the thermal inactivation of β-galactosidase which indicates that the enzymatic activity undergoes a considerable change in conformation during denaturation [71]. The fact that the ∆H° value decreases with the increase in temperature reveals that less energy is required to denature the enzyme at high temperatures [72]. When entropy of inactivation (∆S°) was calculated at each temperature, it showed positive values, which indicates that there is an increase in the molecule randomness or disorder during the exposure to high temperatures. In contrast, negative ∆S values are expected for an aggregation process in which a few intermolecular bonds are formed [73]. Whilst the positive (∆G) values indicate that the inactivation of the enzyme is not a spontaneous reaction. In the present study, there was a decrease in ΔG values with increasing temperatures, which is an indication that the destabilization of the enzyme molecule is more spontaneous and faster [74], however, a lower ∆G° values than the ∆H° values is due to the positive entropic contribution during the inactivation process.

## Conclusion

The medicinal plants encompass natural products such as phytochemicals, used in several medical applications as anticancer, antidiabetic, and antioxidant agents. Traditionally, leaf extracts of JP and CV are considered as promising natural medicinal agents and widely used for the treatment of diabetes mellitus. In addition, the ability of JP and CV extracts to modulate oxidative stress in the body indicated their role in competing the progress of several metabolic diseases such as diabetes mellitus. Furthermore, oral consumption of plants stimulates excretion or decreases the digestion of carbohydrates by inhibiting carbohydrate catalytic enzymes such as β-galactosidase; the enzyme responsible for the metabolism of carbohydrates into glucose in the digestive tract. All these activities may be attributed to the high polyphenolic contents of JP and CV. This work demonstrates the value of JP and CV as sources of metabolites with relevant pharmaceutical potential and essential to better retain enzymatic activity in food biotechnology and products supplemented with heat-sensitive enzymes and to reduced glucose levels.

## Data Availability

The datasets used and/or analysed during the current study available from the corresponding author on reasonable request.

## Abbreviations

IC_50_: The half-maximal inhibitory concentration
DPPH: 1,1-Diphenyl,2-picrylhydrazyl
FRAP: Ferric reducing antioxidant power
GAE: Gallic acid equivalent
RE: Rutin equivalent
Δ*H*: Enthalpy change
